# Febuxostat ternary inclusion complex using SBE7-βCD in presence of a water-soluble polymer: physicochemical characterization, in vitro dissolution, and in vivo evaluation

**DOI:** 10.1007/s13346-023-01496-4

**Published:** 2024-01-07

**Authors:** Wedad Sakran, Mai Abdel-Hakim, Mohammed S. Teiama, Rania S. Abdel-Rashid

**Affiliations:** 1 Pharmaceutics and Industrial Pharmacy Department, Faculty of Pharmacy, Helwan University, Ain Helwan, POB 11795, Cairo, Egypt; 2 Department of Pharmaceutics and Industrial Pharmacy, Faculty of Pharmacy, Galala University, Attaka, 43713 Suez Egypt

**Keywords:** Ternary inclusion complex, Febuxostat, PEG6000, Oral bioavailability, Sulfobutylether-β-cyclodextrin, LC–MS/MS

## Abstract

Febuxostat (FBX), a potent xanthine oxidase inhibitor, is widely used as a blood uric acid-reducing agent and has recently shown a promising repurposing outcome as an anti-cancer. FBX is known for its poor water solubility, which is the main cause of its weak oral bioavailability. In a previous study, we developed a binary system complex between FBX and sulfobutylether-β-cyclodextrin (SBE7-βCD) with improved dissolution behavior. The aim of the current study was to investigate the effect of incorporating a water-soluble polymer with a binary system forming a ternary one, on further enhancement of FBX solubility and dissolution rate. In vivo oral bioavailability was also studied using LC–MS/MS chromatography. The polymer screening study revealed a marked increment in the solubility of FBX with SBE7-βCD in the presence of 5% w/v polyethylene glycol (PEG 6000). In vitro release profile showed a significant increase in the dissolution rate of FBX from FBX ternary complex (FTC). Oral in vivo bioavailability of prepared FTC showed more than threefold enhancement in *C*_max_ value (17.05 ± 2.6 µg/mL) compared to pure FBX C_max_ value (5.013 ± 0.417 µg/mL) with 257% rise in bioavailability. In conclusion, the association of water-soluble polymers with FBX and SBE7-βCD system could significantly improve therapeutic applications of the drug.

**Graphical Abstract:**

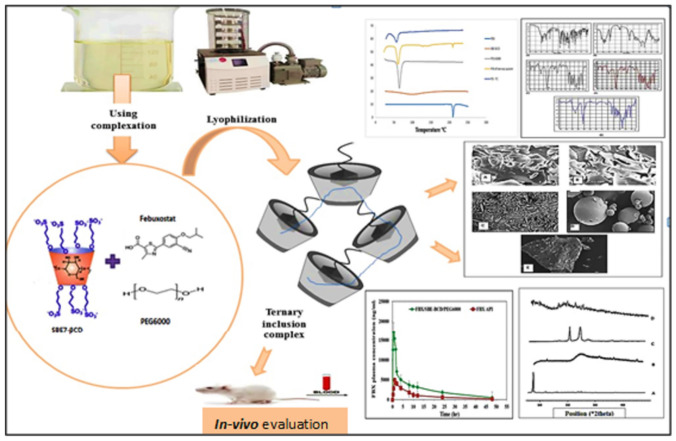

## Introduction

Febuxostat (FBX), an oral non-purine xanthine oxidase (XO) inhibitor, has been prescribed for the treatment of chronic hyperuricaemia and gout in adults since 2009 [1]. In the last few years, FBX has gained a lot of attention and was repurposed for lung cancer treatment and prevention after being identified for its promising cytotoxic properties [2, 3].

FBX is very well known for its limited oral bioavailability (approximately 49%) based on its poor aqueous solubility (practically insoluble) [4], its weakly acidic nature (pKa≈3.08), and its extensive exposure to enzymatic degradation [5, 6]. To combat the major challenge of FBX therapeutic application, which is solubility, several attempts have been implemented and reported in the literature [7]. Among these attempts, drug salt transformation, solid dispersion systems, and co-crystal forms have shown some improvement [8].

Nanocrystal of Febuxostat is a viable approach to enhance drug solubility and its bioavailability [9]; self-nanoemulsifying (SNEDS) have been widely studied for their abundant potential applications that offered greater stability when compared to other lipid-based drug delivery systems. Also, SNEDS improved the solubility and subsequently the oral bioavailability of Febuxostat [10], and nano sponge is spherical microscopic particles comprising interconnecting cavities having the ability to encapsulate a wide range of drug substances, encapsulation of Febuxostat in sustained release nano sponge formulations have successfully been prepared [11].

Inclusion complexes using cyclodextrins have offered a safe and successful approach for improving the solubility of water-insoluble drugs for the past decades. Whereas the cone shape of cyclodextrins can host even more than one drug forming a stable pharmacologically inert complex with significant solubility-enhancing power [12–14]. Its characteristic structure can also improve the stability and permeability of the drug to the biological membranes [15–17].

Sulphobutyl ether β-cyclodextrin (SBE7-βCD) is a derivative of β-cyclodextrin that was rationally designed to reach maximum safety and optimum drug-binding capacity compared to the parent β-CD [18]. Despite being nearly 50 times more soluble than its parent, SBE7-βCD exhibits neither nephrotoxicity nor cytotoxicity due to the ability of the kidney to rapidly excrete ionic compounds [19]. The replacement of hydroxyl in glucopyranose units by an anionic group like sulphate and sulphobutyl-ether also reduces the hemolytic activity of cyclodextrins to negligible values due to its lower ability to derange and solubilize membrane lipids. So, in vivo studies have shown that SBE7-βCD is pharmacologically inactive and well tolerated at high doses [20].

Our research team has previously investigated the effect of forming a binary complex between FBX and SBE7-βCD on the dissolution behavior of the drug. It was found that the freeze-drying technique at a 1:5 ratio of drug to the complexing agent had successfully formed a binary system complex between FBX and SBE7-βCD with nearly double solubility [21]. For further improvement of inclusion efficiency and solubility, the objective of the current investigation is to study the effect of adding a hydrophilic polymer to our previously formed binary complex. It was reported that formulations containing drug-CD complexes in the presence of water-soluble polymers like pectin [22], methylcellulose [23], and polyethylene glycol [24] have proved to be capable of increasing the bioavailability of the formulations and reducing the amount of cyclodextrin used in complex, which is economically beneficial [25, 26]. The polymers increase the wettability of particles, resulting in accelerated dissolution and an increased amount of drug delivered. The type and amount of hydrophilic polymer used are critical factors for the formulation process, as at high concentrations, the viscosity of the medium increases, thus impairing complexation [27–29]. Moreover, it was not reported before the range of ideal polymer concentrations for obtaining ternary complexes, which was very challenging for us.

Thus, the aim of the present study was to design a ternary complex composed of FBX/SBE7-βCD-hydrophilic polymer followed by exploring its physicochemical properties, in vitro dissolution, and *in* vivo oral bioavailability.

## Materials and methods

### Materials

Febuxostat (M. wt., 316.37 g/mol) was kindly donated as a gift by Eva Company for Pharmaceutical Industries, Cairo, Egypt. Captisol®, Sulphobutyl Ether, 7, sodium salt β-cyclodextrin (SBE-βCD, M. wt., 2163 g/mol, purity 99.98%) was kindly supplied from Cydex Inc., USA. Disodium hydrogen phosphate, sodium dihydrogen phosphate, and methanol in the analytical grade were purchased from EL-Gomhoria Company, Egypt. Hydrochloric acid (35%) was purchased from El-Nasr Company, Egypt. Polyethylene glycol 6000 (PEG 6000) and PEG 4000 were purchased from Fluka (Germany). Hydroxypropyl methyl cellulose (HPMC) and polyvinylpyrrolidone (PVP) were also purchased from Sigma Chemical Company (St. Louis, USA). Distilled water was used during the studies. All other chemicals were of HPLC grade.

### Screening of polymers

To select the optimum hydrophilic polymer for the formation of ternary complex, screening of four different water-soluble polymers, namely, HPMC, PEG 6000, PEG 4000, and PVP was carried out to determine their solubilization effect on the complexation efficiency of a previously prepared FBX/SBE7-βCD binary complex [21]. The aqueous solutions of polymers were prepared in distilled water over a concentration range (from 0.1 to 10% w/v) [30]. Accurate weights of FBX and SBE7-βCD were added according to the molar ratio (1:5 of FBX to SBE7-βCD) to the aqueous solutions of polymers. The solutions were shaken using a rotary shaker for 48 h at 37 °C and 100 rpm (thermostatic water bath shaker, RUMO, Egypt). After equilibrium, the solutions were filtered with a hydrophilic cellulose acetate sterile syringe filter (pore size 0.45 µm, diameter 25 mm). Finally, the clear solutions obtained were analyzed spectrophotometrically at *λ*_max_ 315 nm (UV/VIS spectrophotometer, Perkin Elmer Lambda EZ 201, UK) to determine the solubility of FBX in different concentrations of aqueous solutions of polymers. The obtained results were analyzed for FBX solubility improvement, and the selected polymer with a known percentage was utilized for the ternary inclusion complex.

### Preparation of inclusion complexes by lyophilization

Our previously published study selected lyophilization for the preparation of binary complex formation among three other techniques due to its high yield, reasonable inclusion efficiency, and significant dissolution enhancement [21]. The quantities of FBX and SBE7-βCD according to the pre-studied molar ratio (1:5) were dissolved in an aqueous solution containing a certain concentration of the selected polymer, transferred to a conical flask, and allowed to stir for 48 h at 37 °C using a hot plate magnetic stirrer (Jenway 1000, UK). The collected solution was transferred to glass vials and kept frozen for 24 h at − 80 °C in an ultra-cold deep freezer. Thereafter, the samples were freeze-dried using a lyophilizer (Christ Alpha 1–2 LD, Osterode am Harz, Germany) for 24 h to yield a dry powder and stored in airtight containers for further investigation [31].

### Characterization of the ternary inclusion complex

#### Fourier transform infrared spectroscopy (FTIR)

The complex formation was assessed by evaluating the change in peak shape, position, and intensity using a spectrophotometer (FTIR Shimadzu 8400S, Lab Wrench). The spectra of FBX, SBE7-βCD, selected hydrophilic polymer, FBX-ternary inclusion complex, and physical mixture (PM) of raw materials were compared to interpret the spectra. The analysis was performed between 4000 and 400 cm^−1^ and the conformational changes were observed.

#### Differential scanning calorimetry (DSC)

The thermal behavior of FBX, SBE7-βCD, selected hydrophilic polymer, FBX-ternary inclusion complex, and physical mixture (PM) of raw materials were examined using a Shimadzu differential scanning calorimeter including DSC-50 detector with aluminum-sealed pan cell. Samples (4–5 mg) were placed in hermetically sealed aluminum pans and heated in a temperature range of 30 to 300 °C with 10 °C/min increment rate in a nitrogen atmosphere.

#### Surface morphology study

The surface morphology of the different samples of FBX, SBE7-βCD, selected hydrophilic polymer, PM, and FBX-ternary inclusion complex was investigated using a scanning electron microscope. The study was performed using an electron microscope (JSM 6360A, JOEL, Tokyo, Japan). The samples were coated with gold and detected under the microscope at high resolution to reveal the change in morphology.

#### Powder X-ray diffractometry (PXRD)

The crystallinity changes of samples (5 mg each) were determined by PXRD patterns that were recorded using a Diano X-ray diffractometer fortified with Co Kα. The tube operated at 45 kV, and XRD patterns were recorded between the initial and final 2*θ* angle 5° < 2*θ* < 50°.

#### Entrapment efficiency estimation

For the determination of drug content successfully included in the complex, a known amount of the prepared FBX ternary inclusion complex was weighed accurately and transferred into a 50 ml volumetric flask. Thirty milliliters was added of ethanol mixed thoroughly and stirred for 24 h to extract FBX from the inclusion complex at ambient temperature [32]. The volume was made up to the mark with ethanol, and the resulting solution was suitably filtered with a 0.45-µm microfilter for further analysis. The concentration of FBX in the solution was determined using a UV spectrophotometer (UV-1700, Shimadzu, Japan) at *λ*_max_ 315 nm, and drug content was calculated by the following equation:

$$\%\;\mathrm{Drug\;content}=\frac{\mathrm{the\;practical\;concentration}}{\mathrm{the\;theoretical\;concentration}}\times100$$

## Particle size, polydispersity index (PDI), and surface charge (ZP)

Particle size (PS), surface charge (ZP), and polydispersity index (PDI) of using the dynamic light scattering method using a zetasizer 300 HSA (Malvern Instruments, UK), the samples were measured at 25 °C in triplicate. A suitable dilution with distilled water and vortexed was carried out whenever it was necessary [33]. PDI is a measure of the uniformity of particle sizes present in the formulation. A value close to zero (< 0.10) indicates little variability in size (monodisperse), whereas values > 0.10 indicate polydisperse systems. The advantage of a monodisperse system is related to its ability to deliver a consistent amount of compound, as compared to a mixture of polydisperse particles, of different loading capacities [34].

### *In vitro* dissolution study

Simulating oral gastrointestinal conditions, in vitro release profiles of the prepared ternary inclusion complex and pure drug were carried out in 0.1N HCl (pH 1.2) and phosphate buffer (pH 6.8) dissolution media using USP dissolution apparatus II (paddle method). Dissolution in distilled water was also performed for comparative rationale. The studies were carried out using an accurately weighted amount of ternary inclusion complex equivalent to 40 mg of plain FBX in 900 ml of media at 37 °C ± 0.5 °C at a rotation speed of 75 rpm. At preselected time intervals, 5 ml samples were withdrawn, filtered immediately, and replaced with 5 ml of pre-thermo-stated fresh dissolution medium. Quantitative determination was performed by UV spectrophotometer at *λ*_max_ 315 nm for the released quantity of FBX. Each measurement was performed in triplicate, and the graph of cumulative percent drug release versus time was plotted [31].

### *In vitro* release kinetics study

To understand the kinetics and mechanism of FBX release from the ternary inclusion complex, the results were fitted into different models, and the correlation coefficients were determined from regression plots representing zero-order, first-order, Higuchi’s, and Korsmeyer-Peppas models [35]. The following mathematical models were applied respectively:

The equation for zero-order kinetics:

$$Q_t=Q_0-K_0t$$

where, *Q*_0_ = initial amount of drug, *Q*_t_ = amount of drug at time *t*, and *K*_0_ = zero order release constant.

The equation for first-order kinetics:

$$\mathrm{In}\;Q_{\mathit1}=\mathrm{In}\;Q_{\mathit0}-K_{\mathit1}\mathrm t$$

where, *Q*_t_ = the amount of drug released at time *t*, *Q*_0_ is the initial amount of drug, and *K*_1_ is the first-order release constant.

The simplified Higuchi equation:

$$Q_t=K_h{t}^{1/2}$$

where, *Q*_t_ = the amount of drug released at time *t* and *K*_H_ = Higuchi’s constant.

The Korsmeyer-Peppas model relates drug release exponentially to time. It is described by the following equation:

$$Qt=Ktn$$

where, *Q* = the amount of drug discharge in time “t.” *K* = rate constant. *n* = release exponent. The value of *n* indicates the drug release mechanism.

### Effect of storage study

The samples were stored in a powder form in a dissector, and stability studies were carried out at 5 °C ± 3 °C (refrigerator) and at a room temperature (RT) of 25 °C ± 2 °C, and relative humidity of 45 ± 5% RH for a period of 6 months. Periodically, samples were withdrawn to be examined for the physicochemical stability of the complex.

### *In vivo* evaluation of Febuxostat ternary inclusion complex

To explore the efficacy of the formed FBX-ternary inclusion complex on boosting the drug oral bioavailability, in vivo pharmacokinetic parameters of orally administered complex were determined with respect to the plain drug (FBX). The study procedure was approved by the Animal Ethics Committee of the Faculty of Pharmacy, Helwan University, code no. 06A2022.

#### Study design

Eighteen Wistar rats 200 − 250 g ± 20 g in weight each, were divided into three groups (*n* = 6 per group) and participated in a parallel group study. All rats were maintained in a light-controlled room at a temperature of 22 °C ± 2 °C and a relative humidity of 55% ± 5% RH. All groups were fasted overnight (12 h) with free access to water before the experiments. The first group was assigned as a negative control group, while the second group was treated with a single oral dose of pure drug equivalent to 8 mg/kg of FBX that was dispersed in 3 ml of water. The third group received 3 ml of an aqueous solution of ternary inclusion complex containing an equivalent amount of FBX with the same mentioned dose [36].

## Blood sampling

Blood samples (1.5 ml) were obtained from the orbital venous plexus of the rats using a smaller needle, collected in screw-capped heparinized tubes, and immediately centrifuged at 5000 rpm for 10 min for the separation of plasma. The blood samples were withdrawn on the following time schedules: 0.25, 0.5, 1, 1.5, 2, 4, 8, 12, 24, and 48 h post-dose of two treatments. The separated plasma was kept in screw-capped tubes via micropipette and frozen at − 80 °C until assayed.

## Determination of FBX concentration in plasma

A rapid, simple, and highly sensitive LC–MS/MS method has been developed and validated for the quantification of FBX in the presence of cilostazole as an internal standard (IS). An integrated system Shimadzu controller CBM20Alite, containing a pump Shimadzu LC20AT, an auto-sampler Shimadzu SIL20A, and a degasser was used for the study. The analyte and internal standard were separated on Zorbax SB-C18 (75 × 4.6 mm, 3.5 µm) analytical column with an isocratic mobile phase of 80% acetonitrile and 20% of 0.1% formic acid in water at a 1 ml/min flow rate. The autosampler temperature was maintained at 4 °C, and the pressure was maintained at 25 MPa. The injection volume was 20 µl. Quantification was accomplished with MS–MS detection in positive ion mode for the analyte and the IS using a triple quadrupole LC–MS/MS mass spectrometer API 3200 equipped with a TurboIonSpray interface at 550 °C. Multiple reactions monitoring (MRM) was used to display the precursor to product ion transition of 317.1 → 261.1 for Febuxostat and 370.315 → 288.2 for the internal standard (IS). Dwell time was set at 300 ms. The analysis data was created with the software version 1.6. A plasma sample (0.5 ml) was mixed with 4 ml of ethyl acetate in plastic tubes and vortexed. The organic layer was evaporated in a vacuum concentrator and reconstituted in 0.5 ml of the mobile phase and 100 µl of IS following vortex-agitation for 25 s. The tubes were left to stand at ambient temperature for 20 min. After that, the tubes were centrifuged for 15 min. A 20 µl sample of the clear supernatant fluid was injected in to the column.

## Pharmacokinetics analysis

The pharmacokinetic parameters following the oral administration of the treatments were estimated for each rat in each group. The values of the maximum FBX plasma concentration (*C*_max,_ ng/ml), the time to reach *C*_max_ (*T*_max_, h), the area under the plasma concentration–time curve from time zero to 48 h (AUC_0-48_, ng h/ml), elimination rate constant (*K*_el,_ h^−1^), and elimination half-life (*T*_1/2_, h) were obtained from the individual plasma concentration–time curves.

The values of the *C*_max_ and *T*_max_ were obtained directly from plasma data, while the area under the plasma concentration–time (AUC_0-48_, ng h/ml) was calculated using the trapezoidal rule method.

The elimination rate constant (*K*_el_) was calculated from the slope of the terminal part of the concentration time curve, where the slope = − *K*_el_/2.303 then half-life (*T*_1/2_) was calculated as 0.693/ K_el_. Mean residence time (MRT) was calculated from the equation AUMC_0-∞_ / AUC_0-∞_.

The relative bioavailability (Fr) of FBX ternary inclusion complex was calculated in comparison to the aqueous suspension of FBX using the following equation:

$${\mathrm F}_{\mathrm r}\frac{\begin{array}{c}\mathrm{AUC}\;0-48(\mathrm{FBX}\;\mathrm{ternary}\;\mathrm{inclusion}\;\mathrm{complex})\times\;\mathrm{Dose}(\mathrm{FBX}\;\mathrm{aqueous}\;\mathrm{suspension})\\\end{array}}{\mathrm{AUC}\;0-48\left(\mathrm{FBX}\;\mathrm{aqueous}\;\mathrm{suspension}\right)\times\mathrm{Dose}\;(\mathrm{FBX}\;\mathrm{ternary}\;\mathrm{inclusion}\;\mathrm{complex})}$$

All the obtained pharmacokinetic parameters (*C*_max_, AUC_0-48_, AUC_0-∞_, *K*_el_, *T*_max_, and T_1/2_) were analyzed using the IBM SPSS statistics program version 22. A statistically significant difference was considered at *P* < *0.05.*

## Results and discussion

### Screening of polymers

**Fig. 1:**
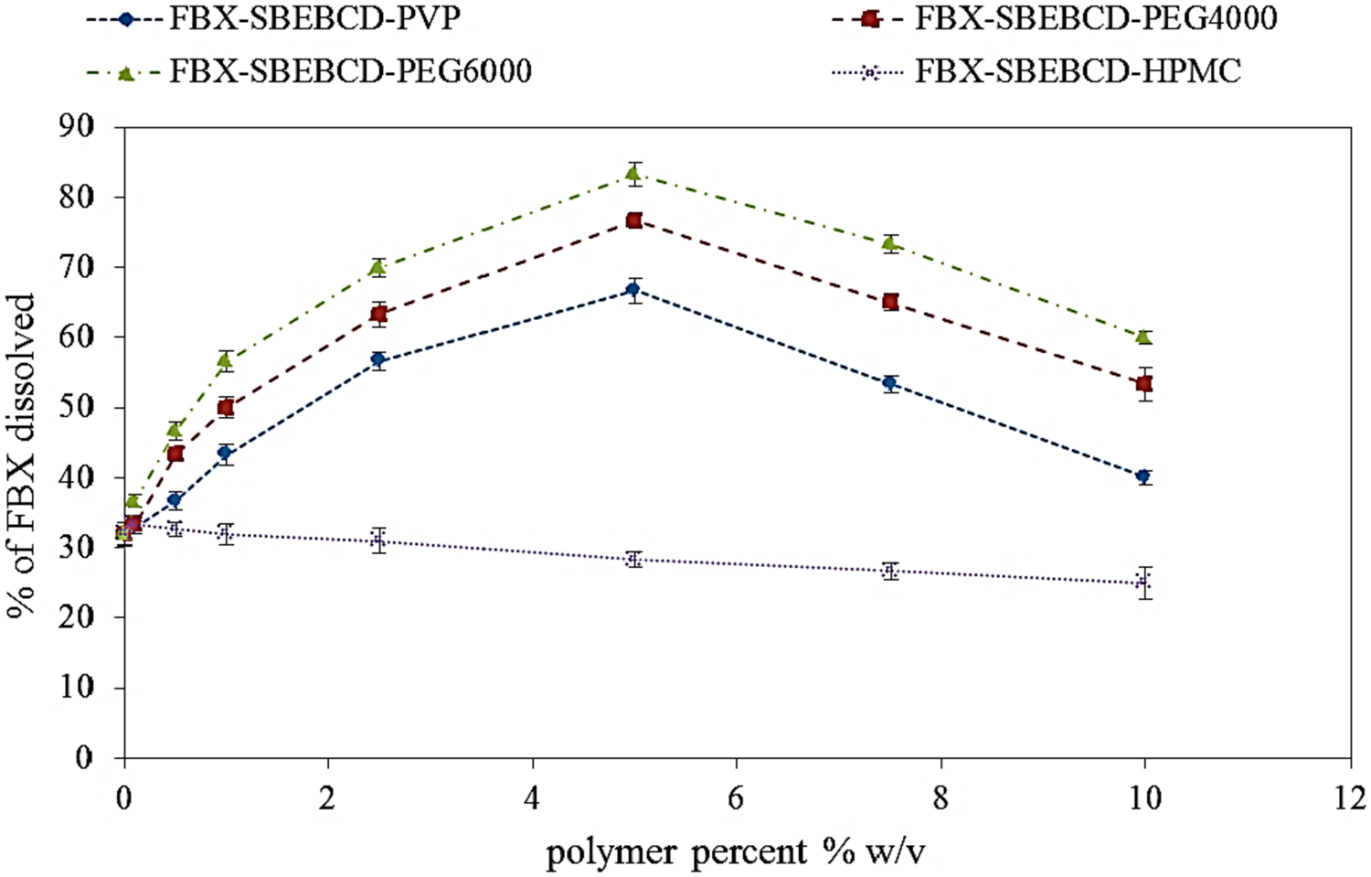
The effect of different hydrophilic polymers on solubilization efficiency of SBE7-βCD with Febuoxstat. FBX present with SBE7-βCD in molar ratio 1:5

As shown in Fig. 1**,** it was found that among the four water-soluble polymers used for the study, 5% w/v PEG 6000 has shown a maximum solubility of FBX 60.1 ± 1.5% compared to the other investigated polymers present in the same percent of PEG 4000, PVP, and HPMC, which showed FBX solubility of 76.6 ± 1.3%, 66.6 ± 1.5%, and 28.3 ± 1.6%, respectively. The investigated polymers PEG 6000 > PEG 4000 > PVP > HPMC increased the solubility of the FBX in ascending order. The increase in the solubility of FBX in the presence of hydrophilic polymers might be related to the increase in complexation efficiency and solubilizing power of cyclodextrins, as reported by several authors [37].

Ternary inclusion complex containing FBX to SBE7-βCD (1:5) molar ratio in the presence of PEG 6000 of 5% w/v showed a higher by 2.6-fold, FBX solubility 83.33 ± 1.6% compared to 32 ± 2.36% from our previously studied binary system. This result was in agreement with a report that showed the addition of PEG 6000 to cyclodextrin solutions resulted in higher solubility and dissolution rate of ternary complexes in comparison to binary complexes, suggesting a significant enhancement in the complexation efficiency between silymarin-βCD [38].

It was obvious from the results that using HPMC and PVP in the formation of ternary inclusion complexes decreased the solubility of FBX. The results mentioned were similar to those recorded by Ammar et al*.*, who found that ternary inclusion complex of glimepiride-SBE-βCD in a molar ratio of 1:3 in the presence of HPMC and PVP 5% w/v showed a decrease in the solubility and dissolution rate of glimepiride [39]. This could indicate a kind of interaction between these hydrophilic polymers and SBE7-βCD, resulting in the formation of polyrotaxanes, wherever many cyclodextrin molecules are threaded onto a linear hydrophilic polymer. Such interaction between CDs and polymers will decrease the ability of CDs to form complexes with the drug [40].

On the basis of the above results, a ternary inclusion complex containing FBX-SBE7-βCD in a (1:5) molar ratio in the presence of 5% w/v PEG 6000 was selected for further investigation.

### Characterization of ternary inclusion complex

#### Fourier transform infrared spectroscopy (FTIR)

**Fig. 2:**
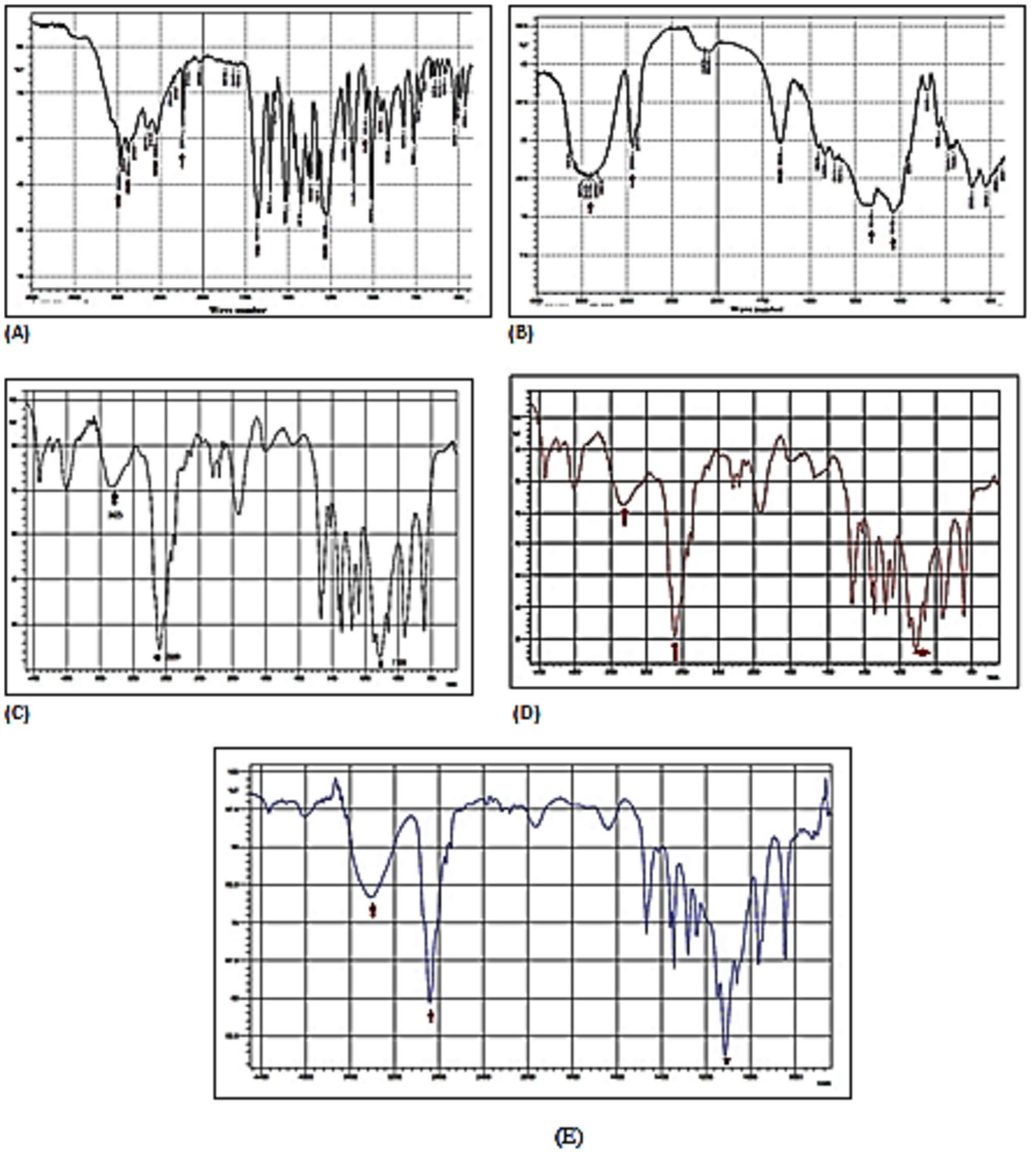
Fourier transform infrared spectra of **A** SBE7- βCD, **B** FBX, **C** PEG 6000 **D** PM of the ternary system, and **E** FD-ternary inclusion complex

The IR spectra for FBX, SBE7-βCD, PEG 6000, a physical mixture of ternary system, and freeze-dried ternary inclusion complex were recorded in Fig. 2. The characteristic stretching peaks of FBX were 2962.66 cm^−1^ and 2873.93 cm^−1^ (alkane-CH group), 2546.04 cm^−1^ (hydroxyl), 2233.87 cm^−1^ (C≡N nitrile stretch), 1681.93 cm^−1^ (C = N stretching of thiazole ring), and 1276.88 cm^−1^ (ether). FTIR spectrum of SBE7-βCD showed characteristic peaks at 3417.86 and 2939.52 cm^−1^, because of the O–H and C–H stretching vibrations and C–O stretching at 1412 cm^−1^. In addition, peaks at 1651.07, 1161.15, and 1041.56 cm^−1^ correspond to H–O–H bending of water molecules attached to CD, C–O, and C–O–C stretching of glucose units, respectively. The spectrum of PEG 6000 showed characteristic peaks at 3425 cm^−1^ (O–H stretch), at 1109 cm^−1^ (C–O–C stretch), and at 2889 cm^−1^ (C-H stretch). However, the physical mixture (PM) spectrum showed a little change, freeze-dried ternary inclusion complex (FD-TC) spectrum showed the disappearance of most characteristic bands of FBX, and the significant low intensity in IR bands of PEG 6000 suggesting complexation of FBX in the presence of SBE7-βCD. Generally, hydrophobic drug molecules have a greater affinity for the cyclodextrin cavity when they are in water solution [41, 42]. Physical mixture compared to the freeze-drying method supplied lower energy to molecules during preparation which may be insufficient to initiate the collision between molecules [43], but in the case of freeze-drying methods, inclusion complex formation took place at the molecular level, and the energy required for the collision of molecules FBX and SBE7-βCD is supplied from heating and stirring during preparation [44].

## Differential scanning calorimetry (DSC)

**Fig. 3:**
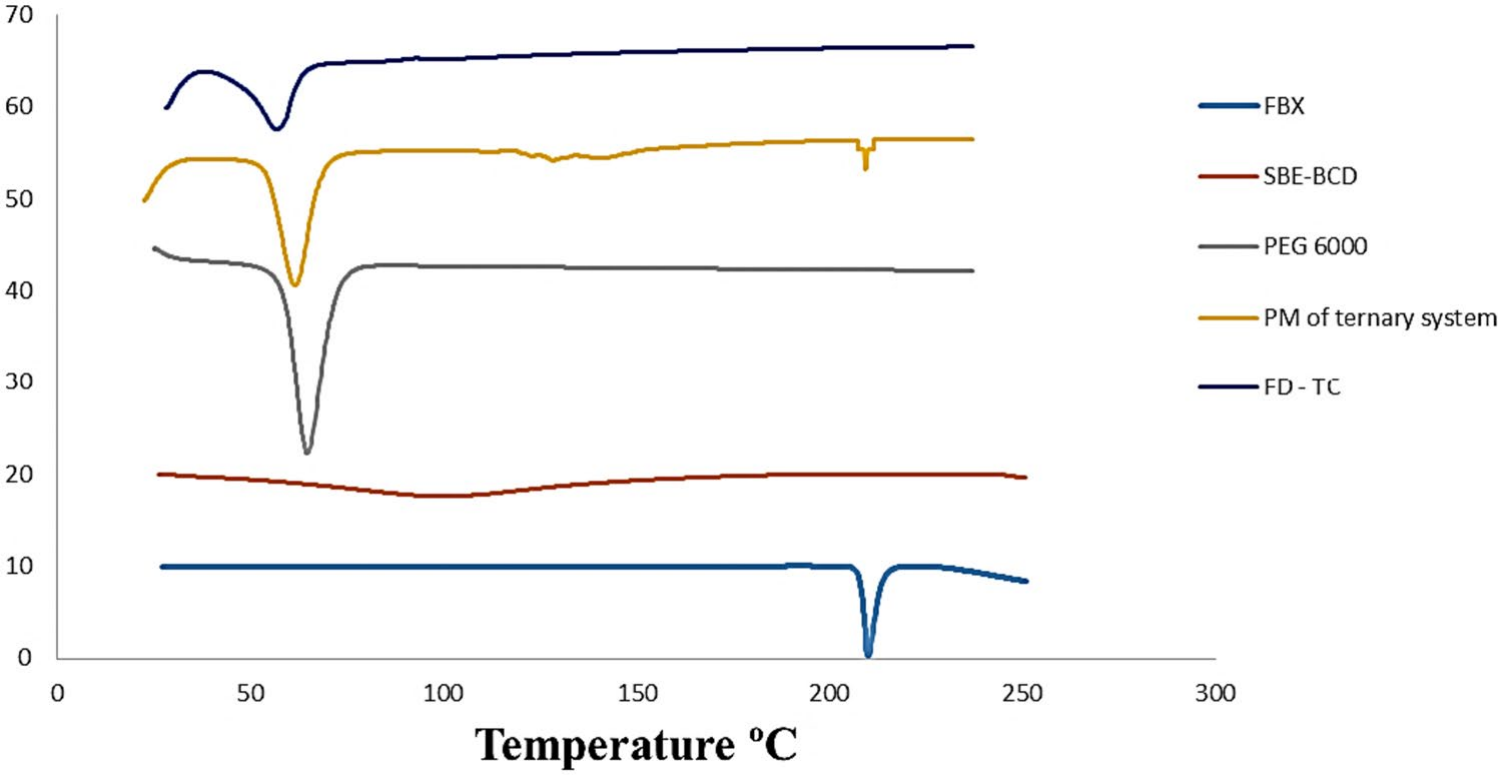
DSC graphs of FBX, SBE7-βCD, PEG 6000, physical mixture of ternary system, and freeze-dried ternary inclusion complex

To confirm the inclusion complex formation, DSC curves may be a useful tool because the absence of an endothermic peak corresponds to the melting of the drug molecule, which indicates the formation of the complex [45]. Figure 3 showed DSC graphs of FBX drug, SBE7-βCD, the physical mixture of the ternary system, and the freeze-dried ternary inclusion complex. The DSC graph of FBX was characterized by a sharp endothermic peak at 209.3 °C [46], corresponding to its melting point, while the SBE7-βCD exhibited a distinctive broad peak at 99.69 °C [47]**.** In the graph of PEG 6000, a sharp peak at 64.6 °C was associated with the melting endotherm of PEG [48].

Consequently, the DSC diagram of the physical mixture of the ternary system showed a shifted peak of SBE7-βCD at 120 °C, an attenuated peak at 209.3 °C for FBX, and a low-intensity peak at 61.5 °C for PEG 6000. On the contrary, the pattern of the ternary inclusion complex FD-TC showed the absence of a distinct peak of FBX and shifted the thermal peak of PEG 6000 at 56.68 °C, as these may indicate the complete incorporation of FBX and ternary inclusion complex formation [49].

## Surface morphology

**Fig. 4:**
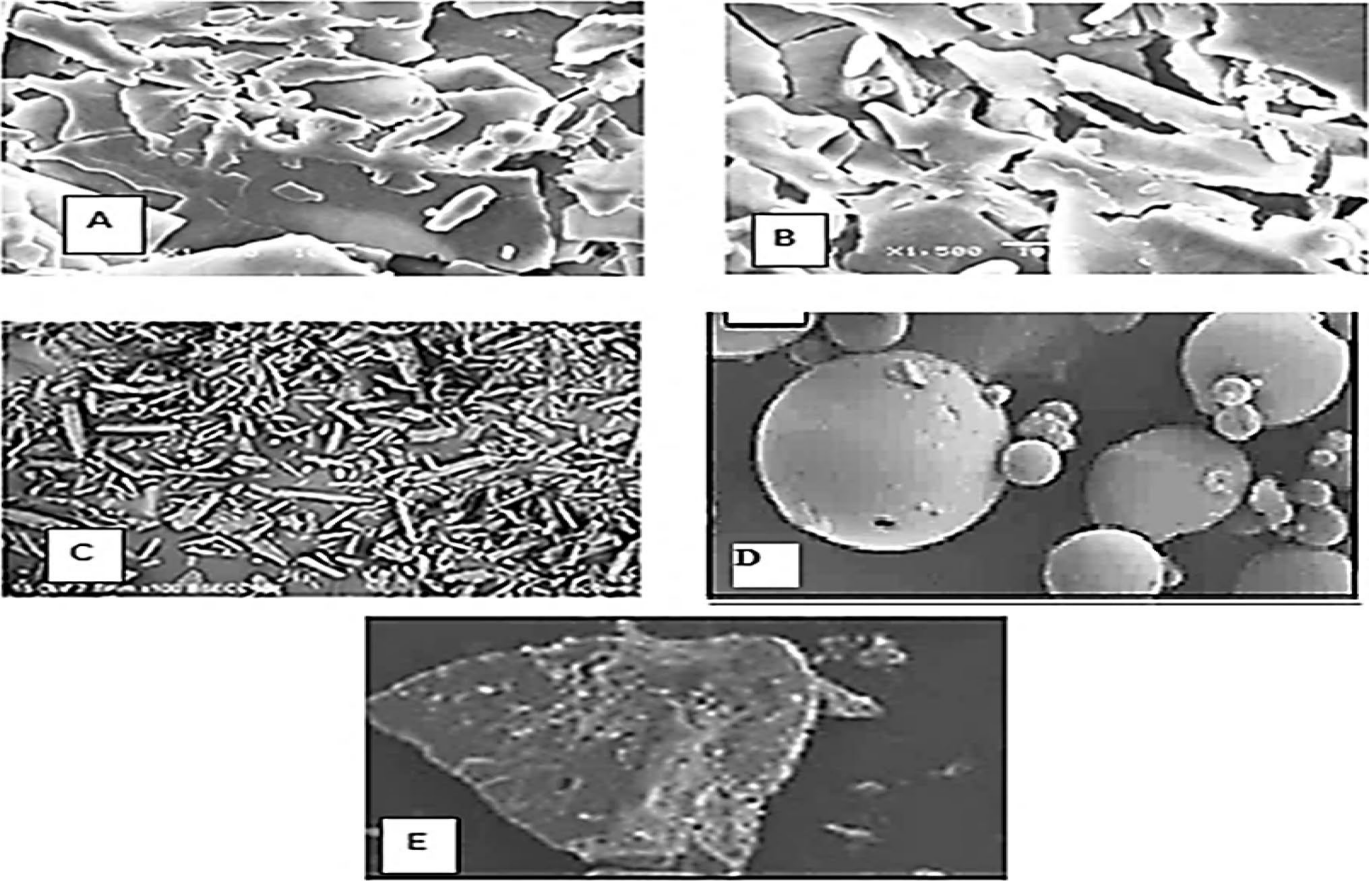
Scanning electron micrographs of ternary inclusion complex (**A**, **B**), FBX (**C**), SBE7-βCD (**D**), and PEG 6000 (**E**)

In Fig. 4, SEM photographs of FBX, PEG 6000, PM, and FBX ternary inclusion complex visualized the morphological changes of those particles. The drug appeared as discrete particles, rectangular needle-shaped indicating its crystalline nature and SBE7-βCD appeared as round, oblong in shape, while the PEG micrograph showed crystals of irregular shape. The specific morphological characteristics of SBE7-βCD and FBX no longer existed in the SEM micrograph of the ternary inclusion complex and the presence of irregular pieces of amorphous aggregates suggested successful complex formation between FBX and SBE7-βCD in the presence of PEG 6000. Thus, altered particle shape and amorphous aggregates might be responsible for improved drug solubility and dissolution rate of FBX [50].

Further freeze-drying technique had contributed to the formation of whole amorphous-natured complexes resulting in fast drug release from the complexes and the presence of a single phase.

## Powder X-ray diffractometry (PXRD)

**Fig. 5:**
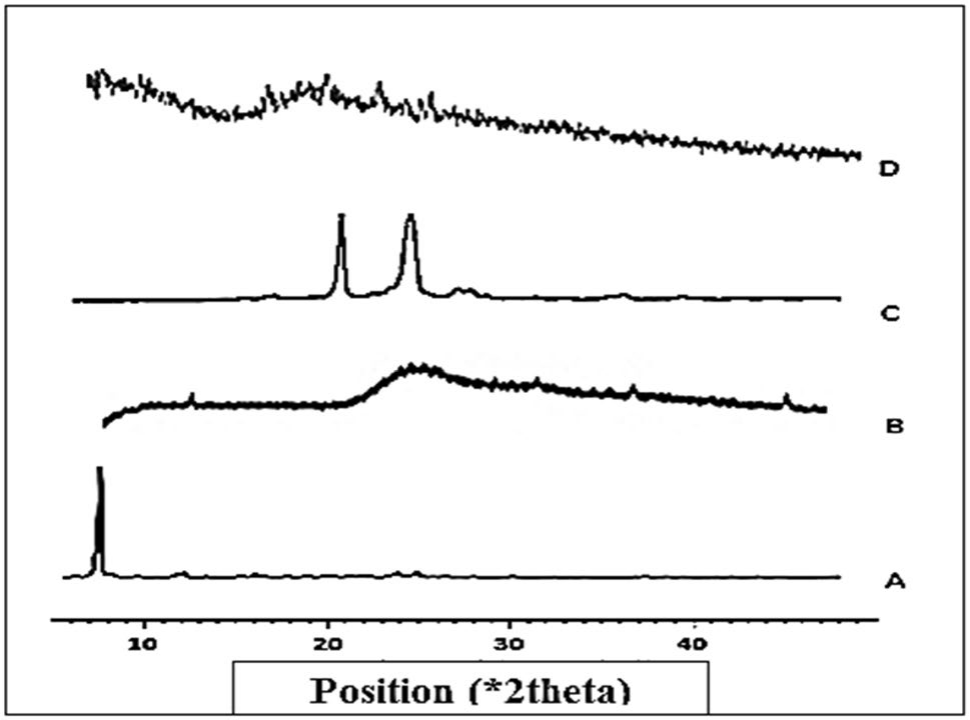
XRD spectra of A) FBX, B) SBE7-βCD, C) PEG 6000, and D) freeze-dried ternary inclusion complex

The PXRD patterns allow examination of the medium- and long-range ordering of materials, which is a useful method to confirm the formation of inclusion complexes. The XRD pattern of FBX in Fig. 5** A** showed characteristic diffraction peaks at 7.2, 12.8, 25.8, and 26.1° 2*θ*, which revealed its crystalline nature, while the X-ray pattern of SBE7-βCD in Fig. 5** B** revealed a halo pattern, indicating its amorphous nature. In Fig. 5** C,** PEG 6000 showed peaks with the highest intensity at 2*θ* of 19.6, 23.4, and 27.1. The XRD pattern of the freeze-dried ternary inclusion complex in Fig. 5** D** showed a halo pattern, with no characteristic peaks of FBX indicating an amorphous form of the powder. These changes can be interpreted as the incorporation of FBX in SBE7-βCD and PEG 6000. These observations were consistent with previous reports by Lateh et al*.* [51].

## Drug content, particle size, and polydispersity index (PdI)

**Fig. 6:**
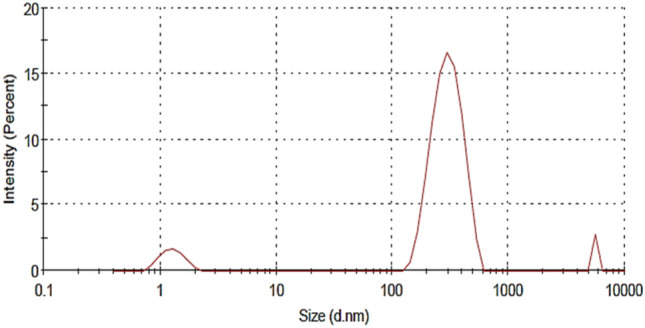
Particle size of FD-ternary inclusion complex

The drug content of the freeze-dried ternary inclusion complex was found to be 83.33 ± 1.6%. The size of FD-TC of FBX-SBE7-βCD in the presence of PEG 6000 showed multicomponent of triple modal size of different populations, which existed as inclusion complexes of 1.3 ± 0.3 nm in diameter with an intensity of 8.2%, small and large inclusion complex aggregates formation of 305.7 ± 87.7 nm and 5143 ± 516.6 nm in diameter with an intensity of 90% and 1.8%, respectively, as showed in Fig. 6. These results were consistent with previous reports by Ibolya et al. [48] that reported the size of ternary Asiaticoside/SBE7-βCD/P407 complex showed multicomponent of trimodal size distributions (5.17, 31.76, 3245 nm) which existed as inclusion complexes, small and large aggregates formation, respectively.

In our study, the PdI of the prepared freeze-dried of ternary system was 0.545 ± 0.05 that reflected particles homogeneity [52]. It was believed that the higher the zeta potential value, the greater the stability for the nanoaggregate system. The zeta potential of the freeze-dried inclusion complex was − 24 ± 2.4 mV. The presence of this charge on the surface of the ternary complex belongs to the anionic nature of SBE7-βCD molecules [53]. Also, it was suggested that the stabilization is regulated by the steric hindrance effect of hydrophilic polymer [54].

### In vitro dissolution studies

**Fig. 7:**
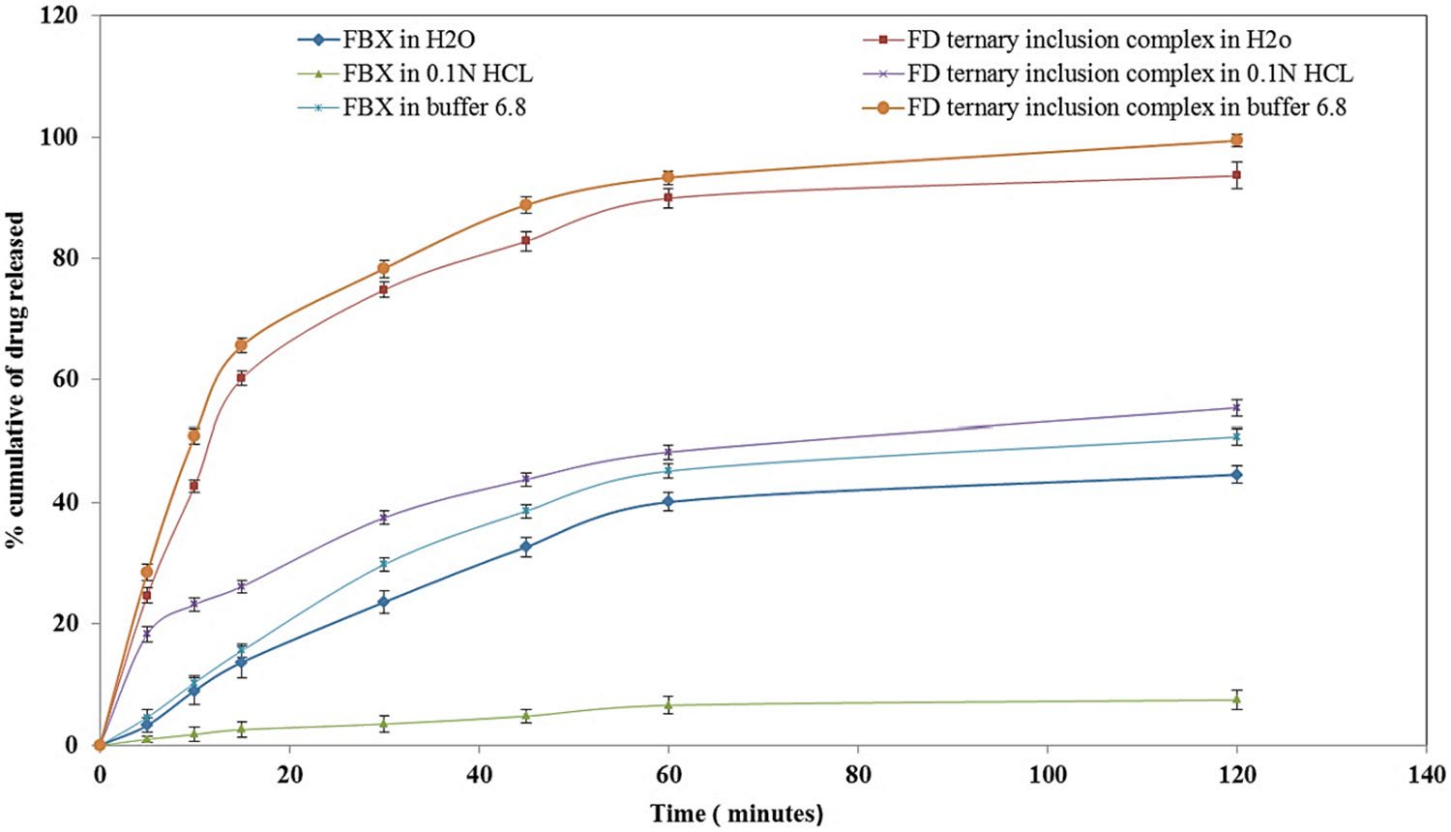
%Cumulative drug release versus sampling time of FBX drug and FD ternary inclusion complex in phosphate buffer pH6.8, 0.1N HCl, and distilled water

It was evident from the data that the ternary inclusion complex served a better dissolution profile and drug release than FBX drug in all the dissolution media as shown in Fig. 7. At 120 min, freeze-dried ternary inclusion complex illustrated 99.4 ± 1.3%, 55.4 ± 0.95%, and 93.6 ± 1.6% drug release in phosphate buffer, 0.1N HCl, and distilled, respectively, which was significantly (*P* < 0.05) higher than FBX drug. The higher dissolution rate of the ternary system can be attributed to the hydrophilic polymer-assisted enhanced complexation in the ternary system, increased drug particle wettability, and reduction of the crystallinity of drug molecules [55].

The possible reason for the difference of sample dissolution rate in different media could be explained by the Henderson-Hasselbalch equation [56]. As the equation indicated, when the pH of the dissolution medium increases, the solubility of the weak acid drug (FBX) increases, and the enhancement of solubility could lead to an increase of dissolution as the Noyes-Whitney equation [57] showed. Besides, the pKa of FBX is around 3.08; therefore, FBX is poorly soluble in water, which explained its low dissolution rate in water. While freeze-dried ternary inclusion complex exhibited a higher dissolution rate regarding to the hydrophilicity of carriers (CDs and hydrophilic polymer).

We fitted the release data using various release models. The resulting correlation coefficients (*r*^2^) of FBX release from freeze-dried ternary inclusion complex were in agreement with the first-order kinetics process, which could be ascribed to passive diffusion [58]. The results were in agreement with the prior investigations performed by Dua et al. that showed the release kinetics of the best formulations of aceclofenac with a β-cyclodextrin molar ratio of 1:2 was observed to follow the first-order release kinetics [59].

Febuxostat in vitro release kinetics from the prepared ternary inclusion complex were studied by applying the Korsmeyer-Peppas model to the release data up to 60%. The ternary complex had a (*n*) value of 0.994, indicating a supercase-II transport in which the release is ruled by the macromolecular relaxation of the polymeric chains, signifying of a combination of diffusion and erosion mechanisms controlling FBX release [60].

### Effect of storage studies

The ternary complex was subjected to stability studies for 6 months at different time intervals (i.e., 1, 3, and 6 months) stored at 5 °C ± 3 °C and at room temperature. It was observed that no significant difference (*P* > *0.05)* was found for percentage drug content and particle size at both conditions for 6 months, so it can be concluded that the formulation was stable for a period of 6 months and showing its suitability for storage at both conditions. The data was recorded in Table 1**.**

**Table 1:** The effect of storage condition on FD ternary inclusion complex at different time intervals stored at 5 °C ± 3 °C and room temperature (25 °C)

**Storage condition**	**Time**	**% content of FBX in selected formula**	**Particle size (nm)**
	Fresh sample	83.33 ± 1.6%	305.7 ± 87.7
**At 5 °C ± 3 °C**	3rd month	83.12 ± 0.99%	306.3 ± 65.7
	6th month	82.22 ± 1.01%	307.7 ± 77.7
	Fresh sample	83.33 ± 1.6%	305.7 ± 87.7
**At room temperature (25 °C)**	3rd month	83.01 ± 1.1%	306.8 ± 57.7
	6th month	82.16 ± 1.33%	308.4 ± 87.7

### Plasma concentration–time data

**Fig. 8:**
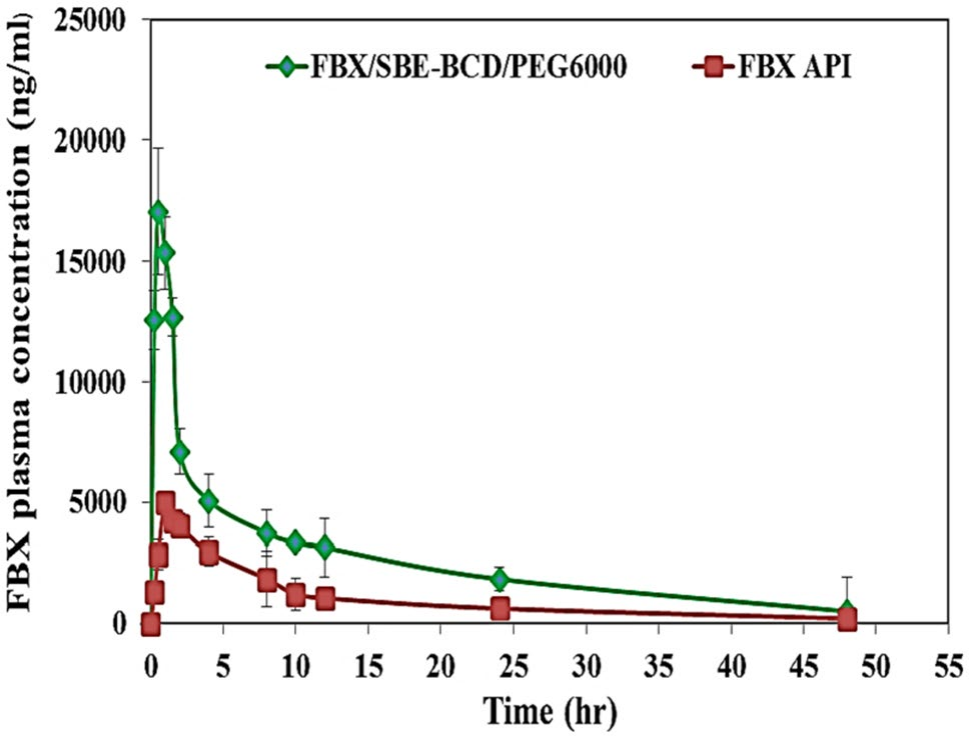
Mean plasma concentration–time curve of FBX (ng/ml) in rats after oral administration of a single dose of the prepared FD ternary inclusion complex and pure FBX aqueous suspension

The mean plasma concentration–time data of Febuxostat following oral administration of a single dose (8 mg FBX/kg) of the prepared ternary inclusion complex and pure FBX suspension to rats were recorded. Figure 8 shows the collective profiles of the mean plasma concentrations of orally administered FBX from the prepared FBX ternary inclusion complex and pure drug suspension.

### Pharmacokinetic parameters

The mean values of the pharmacokinetic parameters are summarized in Table 2. The mean values of *C*_max_ were 17.05 ± 0.811 µg/mL and 5.013 ± 0.417 µg/mL, AUC_0-48_ 126.522 ± 19.9 μg h/mL and 49.22 ± 9.87 µg h/mL, AUC_0-∞_ 143.88 ± 43.2 µg h/mL and 53.84 ± 20.6 μg.h/ml, AUMC_0-∞_ 2086.2 ± 216.2 µg h^2^/mL and 885.71 ± 118.5 µg h^2^/mL, K_el_ 0.053 ± 0.003 h^−1^ and 0.0456 ± 0.001 h^−1^, MRT 14.5 ± 3.35 h and 16.45 ± 4.42 h, and t_1/2_ 12.9 ± 0.74 h and 15.1 ± 3.24 h for FBX ternary inclusion complex and pure drug suspension, respectively. The median values of *T*_max_ following administration of FBX ternary inclusion complex and pure drug suspension were 0.5 h and 1 h, respectively, with interquartile range of 0.25 and 0.5, respectively. The relative bioavailability was found to be 2.57.

**Table 2:** Mean pharmacokinetic parameters of Febuxostat in rats following single oral administration dose (8 mg FBX/Kg) of FBX ternary inclusion complex and pure FBX aqueous suspension

**Pharmacokinetic parameters**	**Mean values (± SD,** ***n* = 6)**	
	**FBX ternary inclusion complex**	**Pure FBX suspension**
***C***_**max**_ **(µg/mL)**	17.05 ± 0.811***	5.013 ± 0.417***
***T***_**max**_ **(h)**	0. 5* (0.25**)***	1* (0.5**)***
**AUC**_**0-48**_ **(µg h/mL)**	126.522 ± 19.9***	49.22 ± 9.87****
**K**_**el**_ **(h**^**−1**^**)**	0.053 ± 0.003****	0.0456 ± 0.021****
**t**_**½**_ **(h)**	12.9 ± 0.74****	15.1 ± 3.24****
**MRT (h)**	14.5 ± 3.35****	16.45 ± 4.42****

*Median; ***P < *0.05*: significant difference; ****P > *0.05*: no significant difference; **Interquartile range; *P-value*: level of significance at 5%

### Statistical analysis of pharmacokinetic parameters

As shown in Table 2, statistical analysis of the pharmacokinetic parameters revealed that the difference between the K_el_, MRT, and t_1/2_ values of both prepared formulation and the pure FBX aqueous suspension was statistically insignificant (*P* > *0.05*). However, there was a significant difference *( P* < *0.05*) between values of *C*_max_, *T*_max_, AUC_0-48_, and AUC_0-∞_ of both prepared formulation and the FBX plain aqueous suspension.

The mean *C*_max_ and AUC_0-48_ in the group administered prepared formulation was 3.4 and 2.57 higher than the group administered FBX plain, respectively.

The statistically significantly (*P* < 0.05) higher *C*_max_, *T*_max_, and AUC_0-48_ that produced by FBX ternary inclusion complex indicated that the oral bioavailability of FBX was improved by embedded FBX in the inner cavity of SBE7-βCD and the formation of rapidly soluble freeze-dried ternary inclusion complex. The increased absorption of the prepared FBX ternary inclusion complex over pure FBX aqueous suspension may be attributed to the improvement in its dissolution and water solubility since FBX could be embedded in the SBE7-βCD cavity and SBE7-βCD has a hydrophilic surface [61]. The pharmacokinetic parameters were in good agreement with those of the dissolution study. The ternary inclusion complex system of FBX showed better bioavailability (2.57-fold increment compered to plain FBX) than the other delivery systems of FBX. Self-nanoemulsifying system for FBX [62], solid dispersions of FBX in the presence of PVP K_30_ and poloxamer188 as combined carriers [46], and FBX nanocrystals [5] showed bioavailability enhancement by twofold, 1.54-fold, and 1.53-fold increments compared to plain FBX, respectively.

The improved solubility and bioavailability of FBX achieved by the formation of ternary inclusion complex of FBX is a promising delivery system for FBX pharmaceutical application.

## Conclusion

In the present study, the preparation of the ternary inclusion complex of Febuxostat-sulfobutylether-β-cyclodextrin was successful in the presence of water-soluble polymer by lyophilization. The prepared ternary inclusion complex was characterized for effective complex formation. In vivo behavior of Febuxostat ternary inclusion complex in a rat model was evaluated and compared to that of pure Febuxostat suspension. In conclusion, the improved solubility and bioavailability of FBX achieved by the formation of FD-TC of FBX make it a promising delivery system for FBX pharmaceutical applications.

## Data Availability

The datasets generated during and/or analyzed during the current study are available from the corresponding author on reasonable request.

## Declarations

### Submission declaration

The manuscript is not under consideration for publication elsewhere.
